# Artificial intelligence standardizes CT-based body composition analysis in breast cacer to address methodological heterogeneity

**DOI:** 10.3389/fonc.2026.1891251

**Published:** 2026-08-03

**Authors:** Wenyue Duan, Ruoyu Wang

**Affiliations:** Department of Radiotherapy, Cancer Center, Affiliated Zhongshan Hospital of Dalian University, Dalian, Liaoning, China

**Keywords:** artificial intelligence, body composition, breast cancer, computed tomography, methodological heterogeneity, sarcopenia

## Abstract

Computed tomography (CT)-derived body composition parameters—including skeletal muscle area, muscle density, and visceral and intermuscular adipose tissue—are robust prognostic and predictive biomarkers in breast cancer. However, their clinical translation is hampered by substantial methodological heterogeneity across studies. Key sources of variability include image acquisition parameters (contrast phase, tube voltage, slice thickness), inconsistent vertebral level selection (L3 versus thoracic levels), the absence of standardized normalization methods, and fragmented diagnostic thresholds for sarcopenia, myosteatosis, and visceral obesity. This narrative review dissects these sources of variability and demonstrates how artificial intelligence (AI), particularly deep learning, provides a transformative solution. AI offers a transformative pathway towards standardization of the analytical workflow—from intelligent slice localization and multi-label tissue segmentation (including challenging compartments such as intermuscular adipose tissue) to the generation of population-specific reference curves and multimodal risk prediction. Open-source platforms such as AutoMATiCA and DAFS Express achieve human-expert accuracy with sub-second processing times, directly addressing reproducibility concerns. By shifting from fixed, population-derived cutoffs to dynamic, individualized reference systems, AI offers a clear pathway toward integrating standardized body composition analysis into routine clinical practice, ultimately advancing precision oncology for breast cancer patients.

## 1 Introduction

Breast cancer remains the most commonly diagnosed malignancy among women worldwide (1), yet the precision of risk stratification and individualized management strategies remains suboptimal. Although body mass index (BMI) has traditionally served as the surrogate marker for adiposity, its limitations in capturing clinically meaningful variations in body composition are now widely recognized. A recent systematic review by Badau et al. demonstrated that BMI alone is insufficient to predict clinical outcomes in patients with hormone receptor-positive, human epidermal growth factor receptor 2-negative (HER2-negative) metastatic breast cancer receiving cyclin-dependent kinase 4 and 6 (CDK4/6) inhibitor therapy, underscoring the need for more refined metrics (2). Indeed, parameters such as visceral adipose tissue (VAT) area and skeletal muscle area (SMA) consistently provide prognostic information superior to BMI (2).

Computed tomography (CT)-based body composition analysis has become the gold standard for quantifying muscle and adipose tissue distribution, offering high precision, the ability to differentiate tissue types by density, and the practical advantage of leveraging routine clinical images (3, 4). Key CT-derived parameters—including the skeletal muscle index (SMI) as a marker of sarcopenia, skeletal muscle density (SMD) as a reflection of myosteatosis, and VAT and subcutaneous adipose tissue (SAT) areas—have demonstrated broad clinical relevance in breast cancer. These metrics inform disease staging, molecular subtype associations, prediction of treatment response and prognosis (5, 6), assessment of chemotherapy and targeted therapy toxicity (7), stratification of surgical complication risk (8, 9), rehabilitation monitoring, and guidance of individualized exercise and nutritional interventions (10, 11).

A typical CT-based body composition analysis workflow comprises image acquisition and preprocessing, localization of reference vertebral levels (e.g., L3, T4/T12), tissue segmentation, and parameter calculation. Artificial intelligence (AI), particularly deep learning, has the potential to substantially advance this workflow by enabling fully automated segmentation, thereby dramatically improving analytical efficiency and facilitating large-scale, multicenter studies. Deep learning architectures such as U-Net and transformer-based models have achieved high accuracy in automatic muscle and fat segmentation, with Dice similarity coefficients exceeding 0.95 and processing times under one second per image (12, 13). However, this technological promise has simultaneously exposed critical methodological inconsistencies that impede clinical translation. Technical parameters—including contrast phase, slice thickness, and tube voltage (14)—along with vertebral level selection, segmentation algorithms, and diagnostic thresholds, vary substantially across studies. The consequence is a body of literature plagued by incomparable results and an inability to establish generalizable clinical guidelines. For instance, although reported sarcopenia prevalence in breast cancer ranges from 32% to 41%, SMI cutoffs vary from 38.5 to 41.0 cm^2^/m^2^, directly affecting patient risk stratification (15, 16). Despite the rapidly growing body of literature on AI-driven CT body composition analysis, a comprehensive narrative review addressing methodological standardization as a prerequisite for clinical translation is lacking. Most published studies focus on isolated technologies or single clinical outcomes, without providing a roadmap to overcome the heterogeneity that currently prevents adoption into routine practice. This narrative review evaluates AI-driven CT body composition analysis in breast cancer from two perspectives. First, we dissect the core methodological issues that create the “standardization gap”: the impact of image acquisition parameters, the technical characteristics and measurement reliability across vertebral levels (with emphasis on L3, T4, and T12), the fragmentation of diagnostic cutoffs, and the validation of AI segmentation techniques. Second, we propose how AI—beyond mere automation—serves as an enabling technology for standardized, scalable, and clinically actionable body composition profiling. By synthesizing current evidence, we seek to provide actionable insights to facilitate clinical implementation and guide future research toward prospective multicenter validation and regulatory readiness.

## 2 Methods

### 2.1 Literature search strategy

To ensure methodological transparency, we conducted structured literature searches to inform this narrative review. Given the focused scope—addressing methodological heterogeneity in CT-based body composition analysis and AI-driven standardization—we performed targeted searches designed to capture key evidence across each thematic area, rather than attempting exhaustive retrieval. Consistent with the narrative nature of this review, we did not perform formal quality assessment or quantitative synthesis.

We searched PubMed, Web of Science, and Embase from database inception to July 2, 2026, restricting results to English-language publications. We defined three thematic domains aligned with the major sections of this review.

Domain 1 (clinical evidence and methodological heterogeneity) aimed to capture studies on CT-derived body composition in breast cancer with a focus on methodological aspects. We combined terms for breast cancer, CT imaging, and body composition parameters (e.g., skeletal muscle, adipose tissue, sarcopenia), with additional emphasis on interobserver variability, reproducibility, standardization, vertebral level selection, and threshold determination.

Domain 2 (AI-based body composition analysis) focused on automated segmentation and analysis, combining terms for artificial intelligence, deep learning, and machine learning with body composition and CT imaging terms.

Domain 3 (clinical deployment and regulatory considerations) targeted clinical translation, combining terms for clinical workflow, picture archiving and communication system (PACS) integration, structured reporting, and implementation with body composition and CT imaging terms.

In addition to database searches, we performed backward citation tracking on landmark methodological references, including the Alberta Protocol and established sarcopenia reference standards. For regulatory and technical information unavailable in peer-reviewed literature—such as U.S. Food and Drug Administration (FDA) 510(k) clearance documents, Conformité Européenne (CE) marking announcements, and technical specifications of open-source software platforms—we performed targeted queries of the FDA public database and developer documentation. We incorporated further references through the authors’ domain knowledge to support specific technical or contextual points.

### 2.2 Study selection and evidence synthesis

We reviewed references identified through the structured searches for relevance to the respective thematic sections. Where multiple studies addressed similar questions, we prioritized those with larger sample sizes, multi-center validation, or prospective design. We explicitly discuss methodological limitations of the cited evidence in the relevant sections. Given the narrative scope of this review, we synthesized findings thematically rather than quantitatively, emphasizing the identification of sources of methodological heterogeneity and the evaluation of how AI-based approaches address these challenges.

## 3 Body composition evidence in breast cancer clinical management

### 3.1 Molecular subtype and clinicopathological features

Body composition phenotypes vary significantly across breast cancer molecular subtypes, with distinct adipose and muscle profiles associated with aggressive disease. In young women, elevated intermuscular adipose tissue (IMAT) independently predicts advanced stage (III–IV) and is associated with aggressive subtypes, including triple-negative breast cancer (TNBC) and HER2-positive disease (17). The visceral-to-subcutaneous fat ratio (V/S ratio) exhibits a particularly strong association with TNBC, whereas the “low fat plus low muscle” phenotype is strongly correlated with this aggressive subtype (18). Adipose tissue distribution and muscle composition each contribute independently to subtype-specific aggressiveness, with IMAT and V/S ratio showing the strongest associations with aggressive phenotypes [odds ratios (ORs) ranging from 2.49 to 9.09]. Key findings are summarized in **Table 1**.

**Table 1:** Summary of key body composition studies in breast cancer clinical management.

Category	Study	Clinical context	Key exposure(s)	Sample size	Key finding	Validation status
Molecular subtype	Costa-Pereira 2025 (17)	Young women (20–40 yr), BC	IMAT	190	High IMAT associated with advanced TNM stage (III–IV) (aOR = 2.49)	Single-center; requires validation
	Vural 2025 (18)	BC	V/S ratio	421	V/S ratio strongly associated with TNBC (OR = 9.09, 95% CI 5.55–14.28); low fat + low muscle: OR = 10	Single-center; requires validation
Racial disparity	Magudia 2021 (19)	General outpatient	Race	12,128	All BC parameters vary by race; Black patients: lower VF	Multi-center; externally validated
	Fumagalli 2025 (20)	Stage II–III BC	Race	3,898	SAT predicts BC-specific mortality only in Black women (HR = 1.24)	Large cohort; validated
Longitudinal change	Mendez 2025 (21)	Early BC (systematic review)	Adjuvant therapy	Multiple	Fat mass ↑ 3.3–9.2%; visceral fat ↑ up to 18.9%; lean mass changes inconsistent	Systematic review; validated
	Jang 2022 (22)	Early BC	TCHP vs AC-T	298	TCHP: ↓SMI 1.6 cm^2^/m^2^ (P < 0.001); AC-T: NS	Single-center; requires validation
	Amitani 2021 (23)	BC	SMI decline ≥3%	141	↓SMI ≥3% during NACT → worse DFS (HR = 3.68)	Single-center; requires validation
	Surmeli 2026 (24)	Stage I–III BC	Diabetes	177	Diabetes: only independent predictor of post-NACT SMI loss (β = −1.42)	Single-center; requires validation
	Rentz 2026 (25)	Early BC	Cachectic phenotype	38 (longitudinal)	84% had clinically-relevant decline; 37% cachectic (muscle + adipose wasting)	Single-center; requires validation
Prognosis	Roberto 2024 (15)	BC (meta-analysis)	Sarcopenia	6,130	OS: HR = 1.33; PFS/DFS: HR = 1.29	Meta-analysis; validated
	Xinyi 2022 (6)	Chinese BC	Sarcopenia; visceral obesity	2,948	Sarcopenia-OS: HR = 2.13; Visceral obesity-DFS: HR = 1.46	Single-center (Chinese); requires external validation
	Jeon 2021 (26)	Adjuvant chemotherapy	IMFD	479	BCSS: HR = 2.95; OS: HR = 2.28	Single-center; requires validation
	Sheean 2021 (27)	ER− metastatic BC	Myosteatosis	152	2-year survival: HR = 1.72	Single-center; requires validation
	Cheng 2024 (28)	Stage II–III BC	B-Score (composite)	3,105	B-Score 3 vs 0: HR = 2.11 (dose-response trend)	Multi-center; validated
	Aktas 2025 (30)	Young women (≤40 yr), BC	High IMAT-C (poor muscle quality)	80 (matched case-control)	Shorter PFS: HR = 2.33 (1.15–4.72)	Single-center; requires validation
	de Lima Bezerra 2024 (29)	Young women (<40 yr), BC	SMI	192	Each 1 cm^2^/m^2^ decrease in SMI → +9% mortality hazard; higher adiposity → higher mortality	Single-center; requires validation
	Yao 2022 (31)	BC with bone metastases	Low psoas density	90	Worse OS: HR = 1.79	Single-center; requires validation
	Jang 2025 (32)	Metastatic BC (meta-analysis)	Sarcopenia	1,472	PFS: HR = 1.17 (NS); OS: HR = 0.99 (NS)	Meta-analysis; validated (null)
	Kim 2025 (33)	HR+/HER2– ABC on CDK4/6i	Low SMD (≤28.7 HU)	247	Worse PFS: HR = 1.84	Single-center; requires validation
	Magudia 2021 (19)	General outpatient (CT screening)	Deep learning-derived z-scores	12,128	SMA z-scores predict 2-year survival (P = 0.04); age/race bias eliminated	Multi-center; externally validated
Toxicity/Efficacy	Hertz 2022 (34)	BC on paclitaxel	Low SMA (T11)	39	↓SMA → ↑Cmax → neuropathy risk; infusion prolongation proposed	Single-center (PK); requires validation
	Roberto 2024 (15)	BC (meta-analysis)	Sarcopenia	6,130	G3–4 toxicity: OR = 3.58	Meta-analysis; validated
	Bas 2023 (35)	Anthracycline-treated patients	Sarcopenia	166	Cardiotoxicity: L3-SMI HR = 3.27; PMI HR = 3.71	Single-center; requires validation
	Toama 2022 (36)	Anthracycline-treated BC	PMI loss	192 (BC subset)	PMI ↓10.5%; larger baseline PMI → lower MACE risk	Single-center; requires validation
	Shachar 2024 (38)	MBC on alpelisib	Low SMD, low VAT	38	Low SMD: hyperglycemia (P = 0.03); Low VAT: rash (P = 0.04)	Single-center; requires validation
	Yaacobi Peretz 2025 (37)	MBC on T-DXd	High SAT, high VAT	48	Dose reduction: SAT OR = 5.34; VAT OR = 5.52	Single-center; requires validation
	Mun 2025 (39)	TNBC on chemoimmunotherapy	SMD (per 5 HU)	144	pCR: OR = 1.67 per 5 HU increase	Single-center; requires validation
	Ding 2025 (40)	NST for BC	SMI, SMD	500	tpCR: SMI (P = 0.008), SMD (P < 0.004); nomogram AUC 0.795/0.714	Single-center; requires validation
	Cebeci 2026 (41)	HR+/HER2– MBC on CDK4/6i	PET/CT-derived sarcopenia	77	Worse PFS: HR = 2.37; outperforms BMI/PNI/PIV	Single-center; requires validation
	Oruc 2025 (42)	MBC on ribociclib	SAT volume; SAT SUVmean	73	SAT volume: HR = 4.96 (P = 0.038); SAT SUVmean: HR = 2.99 (P = 0.016)	Single-center; requires validation
	Cheng et al., 2024 (43)	BC (hypothesis-generating)	Body composition phenotypes (low muscle, high adiposity)	52	Low muscle: ↑CTLA4, ↓pan-AKT; High adiposity: ↑CD3/CD8/CD20 in stroma	Single-center; hypothesis-generating
Surgery/QoL	Nakamura 2020 (9)	Expander-based reconstruction	Low SMI/TATI ratio	157	Each 10% ↓ in SMI/TATI → OR = 2.28 for complications	Single-center; requires validation
	Pittelkow 2020 (8)	Autologous reconstruction	Sarcopenia (SMI < 38.5 cm^2^/m^2^)	103	Sarcopenia → ↑ICU stay (1.5 vs 0.02 d) and ↑hospital stay (8.38 vs 5.49 d)	Single-center; requires validation
	Hehir 2025 (44)	Autologous reconstruction (meta-analysis)	Sarcopenia (PMI or SMI)	1,315 (6 studies)	Haematoma: OR = 1.84; no significant difference in flap loss, infection, re-operation	Meta-analysis; validated
	Moon 2022 (45)	Robotic NSM with IBR	Low SMI/TATI ratio	92	Low ratio → NAC ischemia (OR per unit ↑: 0.94, P = 0.030)	Single-center; requires validation
	Du 2024 (11)	Early BC with sarcopenia	Multimodal intervention (nutrition + exercise)	285	Complications: 4.5% vs 52.6%; Recurrence: 22.7% vs 63.2%	Single-center (intervention); requires validation
	Hanna 2022 (46)	Adults with cancer (meta-analysis)	Low skeletal muscle mass	2,776	Global QoL: SMD = −0.27; Physical functioning: SMD = −0.40	Meta-analysis; validated

ABC, advanced breast cancer; AC-T, anthracyclines and cyclophosphamide followed by a taxane; aOR, adjusted odds ratio; AUC, area under the curve; BC, breast cancer; BCSS, breast cancer-specific survival; B-Score, body composition risk score; CDK4/6i, cyclin-dependent kinase 4/6 inhibitors; CI, confidence interval; Cmax, maximum plasma concentration; CT, computed tomography; DFS, disease-free survival; ER, estrogen receptor; G3–4, grade 3–4; HER2, human epidermal growth factor receptor 2; HR, hazard ratio; HU, Hounsfield unit; IBR, immediate breast reconstruction; IMAT, intermuscular adipose tissue; IMAT-C, intermuscular adipose tissue corrected; IMFD, intermuscular fat density; MACE, major adverse cardiac events; MBC, metastatic breast cancer; NAC, nipple-areolar complex; NACT, neoadjuvant chemotherapy; NS, not significant; NSM, nipple-sparing mastectomy; NST, neoadjuvant systemic therapy; OR, odds ratio; OS, overall survival; pCR, pathologic complete response; PET/CT, positron emission tomography/computed tomography; PFS, progression-free survival; PIV, pan-immune inflammation value; PK, pharmacokinetic; PMI, psoas muscle index; PNI, prognostic nutritional index; QoL, quality of life; SAT, subcutaneous adipose tissue; SMD, skeletal muscle density; SMI, skeletal muscle index; SMD, standardized mean difference; SUV, standardized uptake value; TATI, total adipose tissue index; TCHP, docetaxel, carboplatin, trastuzumab, and pertuzumab; T-DXd, trastuzumab deruxtecan; TNBC, triple-negative breast cancer; TNM, tumor-node-metastasis; tpCR, total pathologic complete response; VAT, visceral adipose tissue; VF, visceral fat; V/S ratio, visceral-to-subcutaneous adipose tissue ratio.

### 3.2 Racial and ethnic disparities

Racial and ethnic differences in body composition have important implications for establishing diagnostic thresholds that are generalizable across diverse populations. However, these differences are multifactorial, reflecting an interplay of biological predisposition, socioeconomic determinants, lifestyle factors, and potential systematic biases in imaging acquisition and interpretation. Using deep learning-based automated analysis in a large outpatient cohort (n = 12,128), Magudia et al. demonstrated that all major body composition parameters vary significantly by race and ethnicity, with Black patients consistently showing lower visceral fat area than White patients (19). Fumagalli et al. further reported substantial racial heterogeneity in the association between adiposity and mortality: SAT predicts breast cancer-specific mortality only in Black women, whereas IMAT is associated with all-cause mortality in both Black and White women, with no significant associations observed in Hispanic or Asian/Pacific Islander women (20). These findings underscore the need for demographically stratified reference curves to improve diagnostic equity and accuracy, ensuring that a one-size-fits-all approach does not lead to risk misclassification in specific populations. Key studies on racial and ethnic disparities are summarized in **Table 1**.

### 3.3 Longitudinal changes during treatment

Breast cancer therapies induce significant, regimen-specific body composition alterations that may carry prognostic implications. A systematic review by Mendez et al. found consistent increases in total and visceral fat mass following adjuvant therapy, with premenopausal women experiencing more pronounced deterioration, whereas changes in lean body mass were less consistent (21). The magnitude of change varies by regimen: TCHP (docetaxel, carboplatin, trastuzumab, and pertuzumab) induces more pronounced SMI decline than AC-T (anthracyclines and cyclophosphamide followed by a taxane) (22). Such changes carry prognostic significance: SMI decline of ≥3% during neoadjuvant chemotherapy (NACT) is independently associated with worse disease-free survival (DFS) (23). Emerging evidence also identifies diabetes as an independent predictor of muscle loss during NACT, suggesting that metabolic comorbidities may potentiate treatment-induced body composition deterioration (24). Furthermore, CT-based phenotyping reveals divergent longitudinal patterns: non-cachectic patients (63%) demonstrate compositional shifts with stable muscle quantity, whereas cachectic patients (37%) exhibit wasting of both muscle and adipose tissue independent of weight loss, suggesting that conventional cachexia definitions may under-detect clinically relevant remodeling in early-stage breast cancer (25). Across these longitudinal studies, a consistent theme emerges: while therapy-induced fat gain is nearly universal, changes in lean and muscle mass are more variable and regimen-dependent; however, when significant muscle loss does occur—whether driven by specific regimens (e.g., TCHP) or comorbidities such as diabetes—it consistently portends worse prognosis. Key findings are summarized in **Table 1**.

### 3.4 Prognostic value

Meta-analyses have established sarcopenia as a consistent prognostic factor in breast cancer, with affected patients facing significantly increased risk of mortality and disease progression (15). This association has been independently confirmed in Asian populations (6) and extends across disease stages, though the magnitude varies considerably—from modest effects in unselected cohorts to stronger associations in specific subgroups (**Table 1**).

Beyond muscle quantity, muscle quality is a complementary—and in some contexts, superior—prognostic indicator. High intermuscular fat density independently predicts worse breast cancer-specific survival (BCSS) (26), and myosteatosis at diagnosis is adversely associated with 2-year survival in estrogen receptor-negative (ER-negative) metastatic disease (27). Notably, composite metrics integrating multiple body composition parameters may enhance risk stratification beyond any single measure. For instance, the B-Score—which combines SMI, subcutaneous adipose tissue index, and SAT radiodensity—demonstrates that the risk of death increases incrementally with the number of unfavorable body composition characteristics (28).

The prognostic significance of body composition varies across clinical contexts. In young women (≤40 years), lower SMI is associated with increased mortality hazard, and specific thresholds for IMAT index, VAT area, and skeletal muscle gauge have been identified (29, 30). In patients with bone metastases, lower psoas major muscle density—rather than muscle area—predicts poorer overall survival (OS), underscoring the importance of muscle quality over quantity in advanced disease settings (31).

By contrast, in metastatic breast cancer, sarcopenia shows no significant association with survival in pooled analyses (32); however, low skeletal muscle density retains prognostic value in patients receiving CDK4/6 inhibitors, and myosteatosis remains prognostic in ER-negative disease (27, 33). These findings suggest that in metastatic disease, muscle quality may be a more relevant indicator than muscle quantity, possibly reflecting the distinct pathophysiology of cachexia in advanced cancer.

Importantly, the consistency of prognostic associations is influenced by analytical choices. Studies employing standardized pipelines and demographically adjusted reference curves (e.g., z-scores) demonstrate more consistent findings than those using fixed cutoffs, suggesting that methodological standardization may reduce between-study heterogeneity (19, 28). Key prognostic studies are summarized in **Table 1**.

### 3.5 Treatment toxicity and efficacy prediction

Body composition parameters independently predict both treatment-related toxicity and response to systemic therapies, offering opportunities for dose individualization. The underlying mechanisms are best illustrated by pharmacokinetic studies: low skeletal muscle area reduces paclitaxel volume of distribution, leading to elevated peak concentrations and increased neuropathy risk—a finding that has informed muscle mass-guided infusion prolongation strategies (34). This mechanistic link between muscle depletion and drug exposure provides a biological rationale for the broad associations observed across therapeutic classes.

#### 3.5.1 Predicting treatment toxicity

Sarcopenia is the strongest and most consistently reported predictor of severe chemotherapy toxicity, particularly for anthracycline- and taxane-based regimens, with meta-analytic evidence confirming a substantially elevated risk of grade 3–4 adverse events (15). Beyond hematologic and gastrointestinal toxicity, sarcopenia also predicts anthracycline-related cardiotoxicity, with both L3-based skeletal muscle index and psoas muscle index demonstrating prognostic value (35). Pectoralis muscle loss during anthracycline-based chemotherapy has similarly been associated with major adverse cardiac events (MACE), suggesting that thoracic muscle metrics may serve as accessible surrogates when abdominal CT is unavailable (36). For targeted therapies, distinct body composition parameters correlate with agent-specific toxicity profiles. High adipose tissue area predicts dose reduction risk with trastuzumab deruxtecan (T-DXd), whereas low skeletal muscle density independently predicts increased toxicity with alpelisib (37, 38).

#### 3.5.2 Predicting treatment efficacy

Body composition parameters also predict therapeutic response, complementing the prognostic associations described in Section 1.4 with a focus on treatment-specific intermediate endpoints. In patients with triple-negative breast cancer receiving neoadjuvant chemoimmunotherapy, higher muscle density independently predicts pathologic complete response (pCR) (39). This association extends to broader neoadjuvant settings: a nomogram integrating low skeletal muscle index and high skeletal muscle density demonstrated good predictive performance for total pathologic complete response (tpCR), suggesting that host factors contribute prognostic information beyond tumor biology alone (40). For targeted therapies, body composition similarly influences treatment outcomes. Sarcopenia detected by positron emission tomography/computed tomography (PET/CT) has been shown to predict progression-free survival (PFS) in patients receiving CDK4/6 inhibitors, outperforming conventional nutritional and inflammatory markers (41). In patients treated with ribociclib, SAT volume and mean standardized uptake value (SUVmean) independently predict outcomes, highlighting the prognostic relevance of adipose tissue quality in targeted therapy settings (42). Mechanistically, body composition phenotypes have been linked to differential expression of immune and phosphoinositide 3-kinase/protein kinase B (PI3K/AKT) pathway proteins in breast tumors—low muscle type is associated with increased cytotoxic T-lymphocyte-associated protein 4 (CTLA4) and decreased pan-AKT expression, whereas high adiposity correlates with increased immune cell markers in the tumor stroma—suggesting that the influence of host composition on treatment response may be mediated, at least in part, through modulation of the tumor microenvironment (43).

#### 3.5.3 Cross-therapeutic patterns

Collectively, these studies reveal several patterns. First, sarcopenia most strongly predicts toxicity for conventional chemotherapy, whereas muscle quality parameters better predict efficacy outcomes for targeted and immunotherapies. Second, distinct body composition parameters are relevant for different therapeutic classes—adiposity for antibody-drug conjugates and PI3K inhibitors, muscle quantity for taxanes, and muscle quality for CDK4/6 inhibitors and immunotherapy. Third, the mechanistic pathways vary: low muscle mass affects drug volume of distribution (driving chemotherapy toxicity), whereas muscle quality may reflect systemic inflammation or metabolic reserve that influences immune-mediated treatment responses. Key studies are summarized in **Table 1**.

### 3.6 Surgical complications and quality of life

Sarcopenia is an independent risk factor for postoperative complications following breast reconstruction, though the magnitude of risk varies by procedure. In expander-based reconstruction, a low SMI-to-total adipose tissue index (TATI) ratio predicts complications, with each 10% decrease in the ratio increasing risk (9). In autologous reconstruction, sarcopenia is associated with increased intensive care unit (ICU) and hospital length of stay, though its impact on flap-specific complications is less pronounced (8). A meta-analysis of autologous breast reconstruction confirmed an elevated risk of hematoma formation in sarcopenic patients, without significant differences in flap loss, infection, or re-operation rates (44). In robotic nipple-sparing mastectomy (NSM) with immediate breast reconstruction (IBR), a low SMI/TATI ratio specifically predicts nipple-areolar complex ischemia (45). Collectively, these findings indicate that while sarcopenia consistently increases overall morbidity across reconstruction types, the specific complications and effect sizes differ by procedure.

Importantly, sarcopenia is a modifiable risk factor. Multimodal interventions combining nutrition and exercise have been shown to significantly reduce postoperative complications and recurrence rates in patients with sarcopenia, highlighting the clinical utility of preoperative sarcopenia screening and early intervention (11).

Regarding quality of life, a meta-analysis demonstrated that low muscle mass is consistently associated with poorer global quality of life and physical functioning, whereas associations with social, role, emotional, and cognitive domains were not significant (46). This pattern suggests that the impact of sarcopenia on quality of life may be primarily physical rather than psychosocial, informing the focus of supportive interventions. Key findings are summarized in **Table 1**.

## 4 Methodological heterogeneity: a barrier to clinical translation

Despite the well-established prognostic value of CT-derived body composition parameters in breast cancer, their integration into routine clinical practice remains limited. A fundamental obstacle is the substantial methodological heterogeneity across published studies, which precludes direct comparison of results and impedes the establishment of universally applicable diagnostic standards. This section examines the key sources of variability in the analytical workflow.

### 4.1 Variability in image acquisition parameters

Unlike research-dedicated imaging protocols, clinically acquired CT examinations are optimized for diagnostic objectives rather than quantitative tissue analysis. This practical reality introduces variability that can significantly impact measurement reproducibility.

#### 4.1.1 Intravenous contrast administration

Intravenous contrast administration represents the most consequential technical factor. Contrast enhancement increases skeletal muscle density (SMD) by approximately 18–20 Hounsfield units (HU) during the portal venous phase compared with non-contrast images, whereas visceral adipose tissue (VAT) area decreases by 7–8% (14). Critically, the classification agreement for myosteatosis between unenhanced and contrast-enhanced scans ranges from fair to moderate (kappa = 0.33–0.64), indicating that diagnostic categorization cannot be directly transferred across contrast phases (14, 47). Nevertheless, recent work has demonstrated that soft tissue measurements from portal venous scans can be adjusted to non-contrast equivalents using linear relationships, with correlation coefficients exceeding 0.90 for muscle and adipose tissue areas (48).

#### 4.1.2 Tube voltage

Tube voltage also influences quantitative tissue measurements. The standard HU thresholds for muscle segmentation (−29 to +150 HU) were originally calibrated for 120 kVp acquisitions. Reducing tube potential to 80 kVp increases SMD by approximately 5–8 HU and modestly alters measured muscle area, necessitating either protocol-specific threshold adjustments or validated conversion factors (14).

#### 4.1.3 Slice thickness

Slice thickness effects are partially mitigated by AI-based segmentation: thin-slice (1.25 mm) versus thick-slice (5 mm) images show strong correlation for automated measures (r^2^ > 0.97), suggesting that deep learning models learn robustness to image characteristics (49).

#### 4.1.4 Emerging technologies

Emerging technologies present both opportunities and challenges. Photon-counting CT and deep learning-based image reconstruction algorithms alter noise characteristics and tissue contrast profiles, potentially affecting HU-based tissue classification (14). Virtual non-contrast (VNC) images from photon-counting CT are comparable to true non-contrast (TNC) images for muscle and parenchymal organ assessment but show significant overestimation of adipose tissue and underestimation of bone. VNC images derived from arterial and portal venous phases do not differ significantly. Therefore, VNC cannot fully replace TNC, particularly for fat and bone evaluation (50). When such technologies are employed, site-specific validation against conventional protocols is strongly recommended.

### 4.2 Inconsistency in vertebral level selection and normalization

The third lumbar vertebra (L3) has emerged as the preferred anatomic landmark for body composition analysis, reflecting its strong correlation with whole-body skeletal muscle and adipose tissue volumes. Derstine et al. established healthy reference values from T10 through L5, demonstrating that skeletal muscle area peaks at L3 in both sexes, supporting its use as the primary measurement site (51). The same investigators subsequently showed that measurements at the mid-vertebral slice versus the inferior aspect of the same vertebra differ significantly, with differences up to 9.3% at L4 in men (52). This finding underscores that sarcopenia cutoffs must reference the specific vertebral slice location from which they were derived.

However, a fundamental limitation of L3-based assessment is its limited clinical applicability in breast cancer patients. Unlike patients with abdominal or pelvic malignancies—who routinely undergo abdominal CT for staging and surveillance—breast cancer patients typically undergo chest CT for preoperative preparation, postoperative review, and radiation therapy planning (53, 54). Per current National Comprehensive Cancer Network (NCCN) guidelines, CT imaging including the abdomen and pelvis is not recommended for asymptomatic patients with early-stage breast cancer, and chest CT commonly does not extend to the lumbar region (53). Consequently, obtaining L3-level CT images is not always feasible for breast cancer patients in routine clinical practice (55). This practical reality represents a major barrier to the widespread clinical implementation of L3-based body composition assessment, as most breast cancer patients lack abdominal CT imaging from which L3 measurements can be derived.

When L3 is unavailable, alternative levels can be employed with appropriate validation. Derstine et al. provided sex-specific reference values and optimal height adjustment formulas for all levels from T10 to L5 (51, 52). For breast cancer patients who typically undergo chest CT for staging and surveillance, thoracic levels offer practical alternatives that are readily accessible from routine imaging. The following thoracic landmarks have been validated in breast cancer cohorts.

#### 4.2.1 The fourth thoracic vertebra

The fourth thoracic vertebra (T4) is the most extensively studied thoracic alternative. Daly et al. demonstrated that T4 and L3 are highly concordant for total and subcutaneous adiposity, intermuscular adipose tissue, and muscle attenuation, but show weaker concordance for VAT and skeletal muscle area (54). A large cohort study of 2,127 patients with stage II–III breast cancer further demonstrated that T4 adiposity independently predicts all-cause and cardiovascular mortality, with hazard ratios comparable to those derived from L3—despite only moderate-to-strong correlations between the two levels. This suggests that T4 measurements may provide complementary prognostic information, possibly due to the proximity of T4 to the breast tumor and heart, rather than serving solely as a surrogate for L3 (Cao et al., 2025). The authors concluded that although T4 and L3 are highly concordant for total and subcutaneous adiposity, fundamental anatomic differences in fat distribution and muscle composition between the thorax and abdomen lead to poorer agreement for VAT and skeletal muscle (54). Nevertheless, the clinical feasibility of T4—which is routinely included in chest CT scans for radiotherapy planning and surveillance—represents an irreplaceable advantage for breast cancer patients who do not undergo abdominal imaging.

#### 4.2.2 The T11 level

The T11 level has also demonstrated utility. Hertz et al. systematically compared seven vertebral levels (T10 through L4) for predicting paclitaxel pharmacokinetics in breast cancer patients, finding that T11 skeletal muscle area provided the optimal model fit (34). Mao et al. demonstrated that the subcutaneous fat index at T11 independently predicts both overall and recurrence-free survival. The authors explicitly noted that “abdominal CT is impractical for patients with breast cancer, making chest CT an essential tool for postoperative surveillance,” and selected T11 precisely because it is routinely included in chest CT scans, capturing key muscle groups and providing reliable adipose tissue data (55).

#### 4.2.3 The T12 level

The T12 level offers an additional thoracic option. Huayan et al. validated an SMI cutoff of 28.01 cm^2^/m^2^ at T12 for Chinese patients with triple-negative breast cancer, with sarcopenia at this level significantly predicting both overall and disease-free survival (56). This work provides a population-specific, clinically applicable threshold for thoracic-level sarcopenia diagnosis, though it requires external validation in broader cohorts.

#### 4.2.4 The L1 level

The L1 level, while technically lumbar, is often included in chest CT protocols that extend to the upper abdomen, making it practically accessible for many breast cancer patients. Chen et al. validated that CT L1-level body composition parameters strongly correlate with L3 measurements (r > 0.7) and predict hematologic toxicity during neoadjuvant chemotherapy (57). Extending this utility, Zhong et al. demonstrated that L1-level parameters on routine chest CT can predict pathological complete response to neoadjuvant therapy (58).

For pectoralis muscle assessment, the T4 level at the sternal angle of Louis has been established as a reliable landmark, with Pecchi et al. demonstrating that pectoralis muscle area on breast MRI correlates strongly with CT-derived skeletal muscle parameters and proposing a cutoff of 20.55 cm^2^ for sarcopenia diagnosis (59). **Table 2** summarizes the utility of different vertebral levels for body composition assessment.

**Table 2:** Vertebral levels for CT-based body composition analysis in breast cancer.

Vertebral level	Chest CT feasibility	Advantages	Limitations	Clinical validation in breast cancer	Recommended use
L3	Not routinely included; requires dedicated abdominal CT	Strongest correlation with whole-body composition; most extensively validated (51) (5)	Not routinely imaged in breast cancer staging/surveillance	Sarcopenia: HR 1.41 for OS (5); Toxicity: OR 3.58 for G3-4 chemotherapy toxicity (15)	Gold standard when available
L1	Partially included (upper abdomen on extended chest CT)	Strong correlate of L3 (r > 0.7); accessible on extended chest CT (57)	Less extensively validated than L3; CT protocol-dependent availability	Strong correlate of L3 (r > 0.7, P < 0.001) (57); SMI < 32.91 cm^2^/m^2^ predicts severe hematologic toxicity (OR not reported) (57); Predicts pCR to neoadjuvant therapy (58)	Preferred upper-level alternative when L3 unavailable and chest CT extends to L1
T12	Routinely included on standard chest CT	Validated cutoff in TNBC; accessible on routine chest CT (56)	Population-specific; requires external validation	Sarcopenia (SMI < 28.01 cm^2^/m^2^) predicts OS (HR 2.885) and DFS (HR 3.121) in TNBC (56)	Thoracic alternative for TNBC; external validation needed
T11	Routinely included on standard chest CT	Optimized for paclitaxel PK (34); accessible on routine chest CT	Limited survival outcome validation	T11 skeletal muscle area optimizes paclitaxel Vd model (34); SFI (cutoff 49.31 cm^2^/m^2^) predicts OS (HR 2.50) and RFS (HR 2.04) (55)	Thoracic alternative for chemotherapy dosing and adipose assessment
T4	Routinely included on standard chest CT	High concordance for TAT/SAT/IMAT (ρ = 0.82–0.83) and muscle attenuation (ρ = 0.84) (54); accessible on routine chest CT	Poor concordance for VAT (ρ = 0.48) and SMI (ρ = 0.58) (54)	T4 adiposity predicts all-cause mortality (intrathoracic: HR 1.35; intermuscular: HR 1.26) and CVD mortality (HR 2.23; HR 2.25) (53); PMI predicts DMFS and OS (84, 107)	Thoracic alternative for adipose tissue and muscle quality assessment

CVD, cardiovascular disease; DFS, disease-free survival; DMFS, distant metastasis-free survival; G3-4, grade 3-4; HR, hazard ratio; IMAT, intermuscular adipose tissue; OS, overall survival; pCR, pathological complete response; PK, pharmacokinetics; PMI, pectoralis muscle index; RFS, recurrence-free survival; SAT, subcutaneous adipose tissue; SFI, subcutaneous fat index; SMI, skeletal muscle index; TAT, total adipose tissue; TNBC, triple-negative breast cancer; VAT, visceral adipose tissue; Vd, volume of distribution; ρ, Spearman’s rank correlation coefficient.

#### 4.2.5 Body size normalization

Body size normalization also lacks consensus. The skeletal muscle index (SMI = muscle area/height^2^) is the most widely reported sarcopenia metric. However, Derstine et al. demonstrated through allometric analysis that height to the power of one (SMA/height) provides optimal height adjustment, whereas SMI retains a significant negative correlation with height and thus systematically classifies taller individuals as sarcopenic (52). The same group developed relative muscle index (RMI) equations that adjust for both height and body mass index (BMI) simultaneously, producing z-scores uncorrelated with body size. These equations enable unbiased comparisons across diverse body habitus and represent a methodological advance over traditional fixed cutoffs (52).

In summary, while L3 remains the gold standard when available, the practical reality that most breast cancer patients undergo chest CT rather than abdominal CT makes thoracic-level alternatives not merely a compromise, but a clinical necessity for translating body composition analysis into routine breast cancer care. T4, T11, T12, and L1 each offer distinct advantages in terms of clinical availability and feasibility, albeit with trade-offs in concordance for specific tissue compartments. Future research should focus on establishing breast cancer-specific reference values and validated clinical thresholds for these thoracic and upper lumbar landmarks, enabling standardized body composition assessment accessible to the majority of breast cancer patients in routine practice.

### 4.3 Fragmentation of diagnostic thresholds

The lack of universally accepted cutoffs represents a major barrier to clinical translation. This fragmentation manifests across multiple dimensions: the choice of SMI threshold, the definition of myosteatosis based on muscle attenuation, the classification of visceral obesity, and the cutoffs used for alternative vertebral levels.

#### 4.3.1 Sarcopenia and myosteatosis thresholds

Sarcopenia prevalence in breast cancer studies ranges from 32% to 41%, yet diagnostic thresholds vary considerably, with SMI cutoffs ranging from 38.5 to 41.0 cm^2^/m^2^ (15, 32). Even greater heterogeneity exists for myosteatosis definition. Unlike sarcopenia—which relies on area-based measurements that are relatively robust to acquisition parameters—myosteatosis is defined using SMD thresholds that are highly sensitive to contrast phase, tube voltage, and reconstruction algorithms (14). The most commonly used myosteatosis thresholds are BMI-stratified (SMD < 41 HU for BMI < 25 kg/m^2^ and SMD < 33 HU for BMI ≥ 25 kg/m^2^), whereas some studies occasionally employ sex-specific cutoffs or uniform HU thresholds (e.g., < 22.5 HU or < 33 HU) (60). The use of different HU thresholds across studies has led to widely varying reported myosteatosis prevalence (11–85% across cancer types) and inconsistent associations with clinical outcomes (60). **Table 3** summarizes the major thresholds, their population contexts, and validation status.

**Table 3:** Diagnostic thresholds for sarcopenia and myosteatosis in breast cancer.

Parameter	Threshold	Population context	Validation status	Key supporting study
Sarcopenia (SMI, L3)				
	< 38.5 cm^2^/m^2^	General population-derived	Multi-study validation; used in prospective cohorts	Prado et al., 2009 (108)
	< 40.0 cm^2^/m^2^	General population-derived	Validated in large breast cancer cohort (n=3,241)	Caan et al., 2018 (5)
	< 41.0 cm^2^/m^2^	General population-derived	Widely used in breast cancer studies; meta-analysis	Rier et al., 2017 (109); Shachar et al., 2017 (110)
	< 40.0 cm^2^/m^2^	Breast cancer-specific (young women)	Single-center retrospective (n=80); requires external validation	Aktas et al., 2025 (30)
	< 28.01 cm^2^/m^2^ (T12)	Population-specific (Chinese TNBC)	Single-center retrospective (n=130); requires external validation	Huayan et al., 2025 (56)
	< 48.6 cm^2^/m^2^ (L3 transverse process)	Breast cancer-specific	Single-center retrospective; requires external validation	Kang et al., 2024 (61)
Myosteatosis (SMD, L3)				
	< 41 HU (BMI < 25)/< 33 HU (BMI ≥ 25)	BMI-stratified	Multi-study validation in metastatic breast cancer; HR 1.72–2.04	Rier et al., 2017 (109); Sheean et al., 2021 (27)
	≤ 28.7 HU (lowest tertile)	Breast cancer-specific (CDK4/6i cohort)	Single-center prospective cohort (n=247); requires external validation	Kim et al., 2025 (33)
	< 37.8 HU	General population-derived	Validated in breast cancer cohort (n=3,241)	Caan et al., 2018 (5)

BMI, body mass index; CDK4/6i, cyclin-dependent kinase 4/6 inhibitors; HR, hazard ratio; HU, Hounsfield unit; SMD, skeletal muscle density; SMI, skeletal muscle index; TNBC, triple-negative breast cancer.

Validation status definitions: Multi-study validation — threshold validated in ≥2 independent studies or included in meta-analyses; Large cohort validation — validated in a single large study with >1,000 patients; Requires external validation — derived from a single-center retrospective study, awaiting independent confirmation.

#### 4.3.2 Population-specific thresholds

Emerging evidence suggests that population-specific thresholds are necessary. Magudia et al. demonstrated that traditional SMI thresholds introduce age and race bias, whereas z-scores derived from demographically adjusted reference curves produce consistent sarcopenia prevalence (35%) across all age and race subgroups (19). Kang et al. proposed a breast cancer-specific SMI cutoff of 48.6 cm^2^/m^2^ at the L3 transverse process level—substantially higher than general population standards—suggesting that cancer populations may require disease-specific reference values (61). For thoracic-level assessment, Huayan et al. validated an SMI cutoff of 28.01 cm^2^/m^2^ at T12 for Chinese patients with triple-negative breast cancer, emphasizing that cutoffs developed at L3 cannot be directly transferred to other vertebral levels (56).

#### 4.3.3 Data-driven cutoff determination

Several strategies have been employed to determine optimal cutoffs when established standards are lacking. These include maximally selected log-rank analysis for survival outcomes (29), receiver operating characteristic (ROC) curves for toxicity endpoints (57), and tertile or median splits for exploratory analyses (17, 43, 62, 63). Although pragmatic, the use of data-driven cutoffs within single studies risks overfitting and limits external generalizability. The field would benefit from consortium-driven efforts to establish standardized, outcome-relevant thresholds stratified by sex, race, cancer stage, molecular subtype, and treatment context.

#### 4.3.4 Clinical implications of threshold fragmentation

The practical consequence of threshold heterogeneity is that systematic reviews and meta-analyses face significant challenges in pooling data across studies. For example, a recent meta-analysis of sarcopenia in metastatic breast cancer reported a pooled prevalence of 41.6% but with substantial heterogeneity driven primarily by differing SMI cutoffs (32). Similarly, the association between sarcopenia and overall survival in metastatic breast cancer was not statistically significant in pooled analysis (HR = 0.99, 95% CI 0.96–1.01); however, this null finding may reflect methodological heterogeneity rather than the true absence of prognostic value (32). Until standardized thresholds are established, clinicians cannot reliably apply research findings to individual patients, and comparisons across institutions or trials remain problematic. Future efforts should prioritize harmonizing thresholds through large-scale, multi-center studies that account for population diversity and imaging protocol variability, with the goal of establishing universally applicable, outcome-relevant cutoffs stratified by sex, race, cancer stage, and molecular subtype.

### 4.4 Summary: the standardization gap

**Figure 1:**
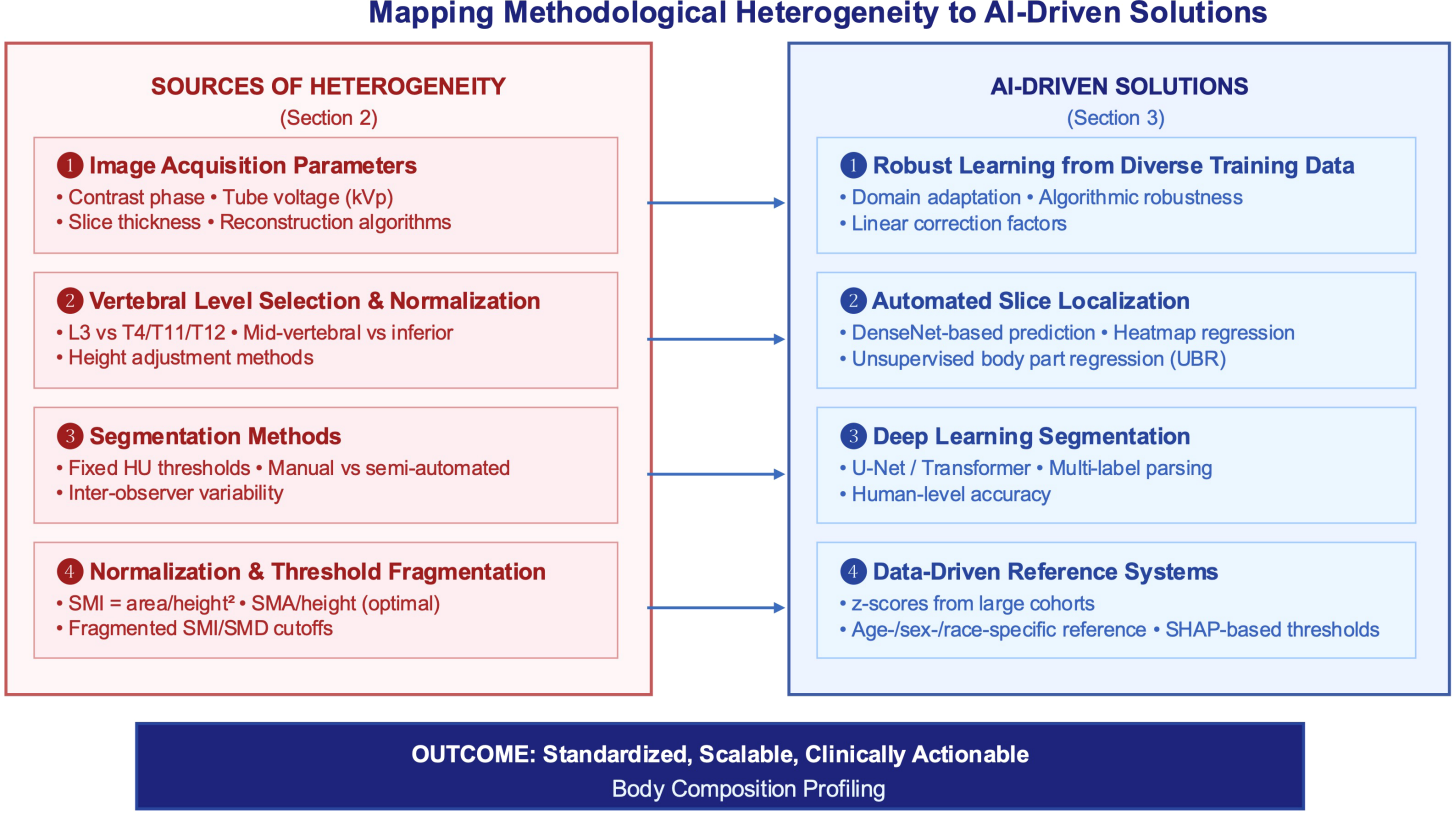
Mapping methodological heterogeneity to AI-driven solutions. The left panel summarizes four major sources of variability in CT-based body composition analysis identified in Section 2: image acquisition parameters, vertebral level selection and normalization, segmentation methods, and normalization/threshold fragmentation. The right panel maps each source to corresponding AI-based solutions discussed in Section 3: robust learning from diverse data, automated slice localization, deep learning-based multi-tissue segmentation, and data-driven reference systems. This mapping illustrates the conceptual framework underlying the review: AI does not merely automate existing methods but enables fundamentally new approaches to standardization. The bottom panel synthesizes the ultimate outcome of this framework. HU, Hounsfield unit; SMI, skeletal muscle index; SMD, skeletal muscle density; SMA, skeletal muscle area; SHAP, SHapley Additive Explanations.

In summary, current CT-based body composition analysis faces multiple interconnected sources of variability: image acquisition parameters, vertebral level and slice selection, body size normalization methods, and diagnostic thresholds. These methodological inconsistencies produce incomparable results across studies and prevent the establishment of evidence-based clinical guidelines. Addressing this standardization gap requires a systematic, technology-enabled solution—which we propose in the following section. **Figure 1** maps the four categories of methodological heterogeneity identified in this section to their corresponding AI-based solutions discussed in Section 3, visualizing the core problem-solution framework of this review.

## 5 AI as an enabler: toward standardized and scalable body composition profiling

Artificial intelligence (AI), particularly deep learning, offers a transformative approach to overcoming the methodological heterogeneity outlined above. Rather than incrementally improving individual steps, AI enables end-to-end standardization of the entire analytical workflow. This section presents AI-driven solutions organized along the analytical pipeline, demonstrating how each addresses a specific source of variability.

### 5.1 Fully automated workflows: eliminating manual variability

Manual segmentation using specialized software (e.g., SliceOmatic, ImageJ) has long been the reference standard, typically following protocols such as the Alberta Protocol (64). Although accurate, manual segmentation is labor-intensive (15–30 minutes per patient), subject to inter-observer variability, and impractical for large-scale or routine clinical applications.

**Figure 2:**
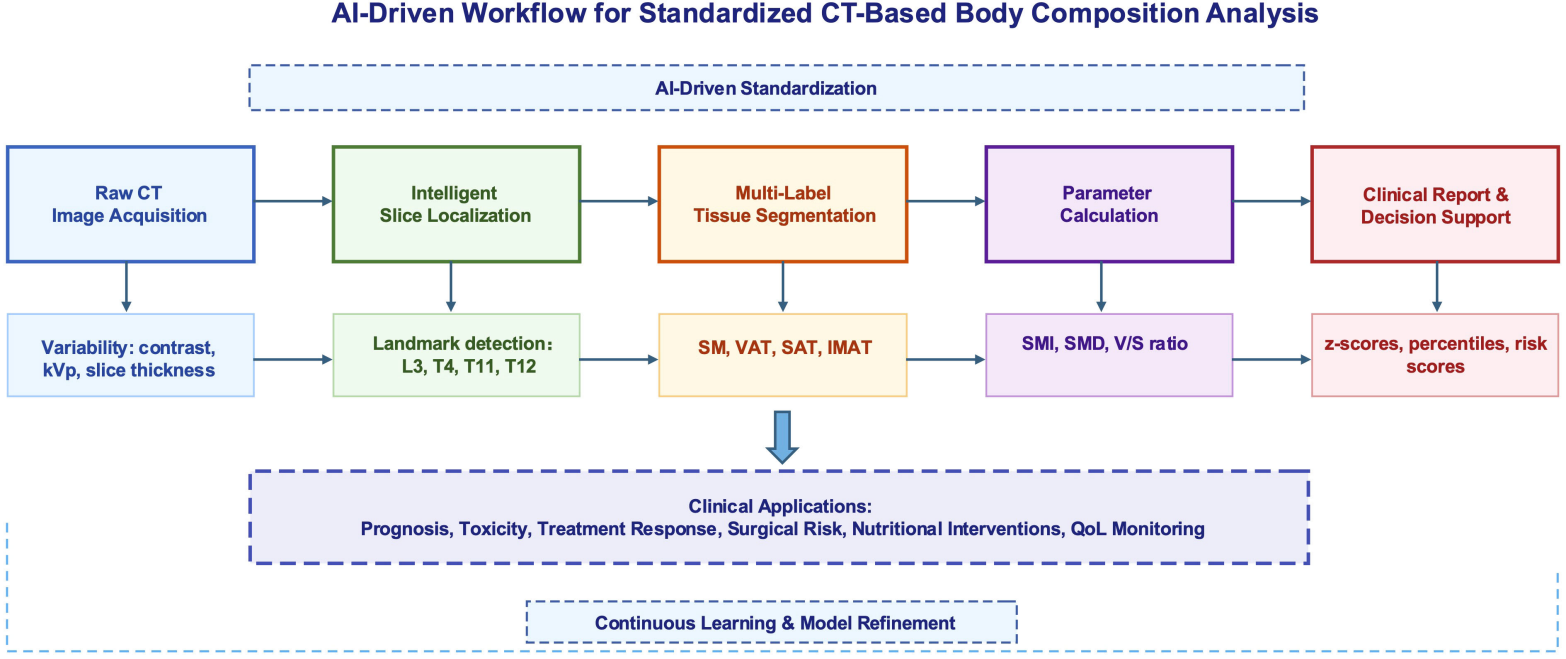
AI-driven workflow for standardized CT-based body composition analysis. The workflow comprises five sequential stages. Stage 1: raw CT image acquisition, with inherent variability in contrast phase, tube voltage, and slice thickness that impacts measurement reproducibility. Stage 2: intelligent slice localization using deep learning to identify reference vertebral levels (L3 as the gold standard, or T4, T11, T12, and L1 as practical alternatives for patients undergoing chest CT). Stage 3: multi-label tissue segmentation distinguishing skeletal muscle (SM), visceral adipose tissue (VAT), subcutaneous adipose tissue (SAT), and intermuscular adipose tissue (IMAT). Stage 4: parameter calculation including skeletal muscle index (SMI), skeletal muscle density (SMD), and fat distribution metrics. Stage 5: generation of demographically adjusted reference curves (z-scores) and structured reports for clinical integration. The dashed arrow from Stage 5 back to Stage 1 represents continuous model refinement through feedback loops. AI automation at each stage (stages 2-4) addresses the methodological heterogeneity described in Section 2, enabling standardized, reproducible analysis. SM, skeletal muscle; VAT, visceral adipose tissue; SAT, subcutaneous adipose tissue; IMAT, intermuscular adipose tissue; SMI, skeletal muscle index; SMD, skeletal muscle density.

Deep learning has revolutionized this workflow. **Table 4** summarizes validated platforms that achieve segmentation accuracy comparable to or exceeding that of human experts, with dramatically reduced processing times. The Data Analysis Facilitation Suite (DAFS) Express platform has been validated in a large cohort of cancer patients and demonstrates strong agreement with manual segmentation; importantly, automated and manual segmentations yielded nearly identical mortality hazard ratios (65, 66). **Figure 2** illustrates the complete AI-driven workflow from image acquisition to clinical reporting, with AI automation addressing key sources of methodological heterogeneity at each stage.

**Table 4:** Validated AI platforms for automated CT body composition analysis.

Platform/algorithm	Architecture	Segmentation targets	Performance (dice)	Processing time	Clinical validation	Key feature	Reference
DAFS Express	Prior shape model + CNN	SM, VAT, SAT, IMAT	SM/VAT/SAT > 0.96; IMAT ~0.77	~0.33 min/case	HR comparable to manual (1.30 vs 1.29 for lowest vs highest muscle tertile)	Clinically validated in >5,000 cancer patients	Salehin (2025) (65); Cespedes Feliciano (2020) (66)
AutoMATiCA	Neural network	SM, IMAT, VAT, SAT	SM 0.983; VAT 0.979; SAT 0.986; IMAT 0.900	~350 ms/scan	—	Open-source; validated across diverse clinical cohorts	Paris (2020) (12)
Swin UNETR	Transformer-based	SM, adipose tissue	SM 0.868; adipose 0.924; outperforms U-Net (0.828 for SM, P < 0.033)	Not reported	—	Outperforms conventional CNN-based models	Yildiz Potter (2025) (68)
Robert et al. multi-label network	Deep learning-based	5 muscle groups + 3 adipose compartments (VAT, SAT, IMAT)	IMAT specifically improved; preferred by radiologists in 93.03% of cases	Not reported	—	Multi-label fine-grained parsing; open-source	Robert (2025) (78)
Deepcatch	Commercial deep learning	SM, fat compartments	0.97–0.99	Not reported	—	Volumetric whole-body segmentation capability	Lee (2021) (111)
TotalSegmentator-based pipeline	Deep learning-based	SM (total), SM (psoas), VAT, SAT	SM (psoas): r=0.776; VAT: r=0.993; SAT: r=0.984 (vs manual)	Not reported	Prognostic for OS	Open-access pipeline; flexible integration	Hofmann (2025) (79)
ABACS (SliceOmatic)	Commercial automated module	SM, VAT, SAT	Jaccard > 0.90	Not reported	Mortality associations similar to manual	Integrated with reference manual segmentation software	Cespedes Feliciano (2020) (66)

ABACS, Automatic Body composition Analyser using Computed tomography image Segmentation; CNN, convolutional neural network; CT, computed tomography; DAFS, Data Analysis Facilitation Suite; HR, hazard ratio; IMAT, intermuscular adipose tissue; OS, overall survival; SAT, subcutaneous adipose tissue; SM, skeletal muscle; U-Net, U-shaped Network (a convolutional neural network architecture); VAT, visceral adipose tissue.

### 5.2 Intelligent slice localization: solving the “which slice” problem

Rentz et al. found that SAT area differed by as much as 79.9%, and skeletal muscle area by 16.8%, between CT slices separated by only approximately 16 mm (67). This magnitude of variability makes manual or semi-automated slice selection a critical source of irreproducibility.

Deep learning-based slice localization directly addresses this issue. Multiple approaches have been developed and validated. DenseNet-based models predict the L3 vertebral level with a median error of 5 mm (19). Heatmap regression achieves 90% localization accuracy with a mean absolute error of 40 mm—within the anatomic range of vertebral length (40–50 mm), making it clinically acceptable for slice selection (68). Unsupervised body part regression algorithms automatically identify T12 through L4 levels, ensuring standardized positioning for key measurement sites (48, 69). Lidia et al. validated an automated L3 selection algorithm with a mean absolute error of 4.0–5.5 mm (10–15% of vertebral height), demonstrating clinical acceptability even in patients with metastatic disease or post-surgical changes (70).

### 5.3 High-precision multi-tissue segmentation: beyond fixed HU thresholding

Traditional CT tissue segmentation relies on fixed HU thresholds—an approach inherently limited by its inability to account for inter-scanner variability, differences in contrast phase, or individual patient characteristics. Deep learning overcomes this limitation by learning tissue-specific intensity patterns, spatial context, and morphological features directly from annotated training data, enabling robust segmentation across heterogeneous imaging protocols.

#### 5.3.1 From threshold-based to learning-based segmentation

Convolutional neural networks now achieve high segmentation accuracy with near-instantaneous processing times—a dramatic improvement over the 15–30 minutes required for manual segmentation (12, 19, 71). The shift from convolutional to transformer-based architectures reflects a broader paradigm change. Vision Transformers (ViT), which rely on self-attention mechanisms to model global contextual relationships, have demonstrated superior robustness in various segmentation tasks, particularly under challenging imaging conditions (72). In CT-based body composition analysis—a task particularly susceptible to parameter-induced variability—transformer architectures such as Swin UNETR have been shown to significantly outperform conventional U-Nets (68). This advantage stems from their capacity to capture long-range spatial dependencies, a critical feature for distinguishing tissues with similar attenuation profiles but distinct spatial distributions.

Building upon these advances, foundation models—large-scale pre-trained models adaptable to diverse downstream tasks with minimal fine-tuning—have opened new avenues for medical imaging. The Segment Anything Model (SAM) and its medical counterpart, MedSAM, demonstrate promising zero-shot segmentation capabilities for abdominal organs in CT imaging (73, 74). However, adaptation to medical imaging remains challenging: while larger organs with clear boundaries achieve high Dice scores (>0.82), performance declines substantially for smaller or low-contrast structures, underscoring the need for task-specific refinement before clinical deployment (73).

Complementary to architectural innovations, self-supervised learning (SSL) has emerged as a strategy to mitigate the annotation bottleneck. By pre-training on large-scale unlabeled CT volumes and fine-tuning on limited annotated data, SSL achieves competitive segmentation performance with substantially fewer labeled examples while enhancing robustness to imaging protocol variations (75, 76). This is particularly relevant for body composition analysis, where manual annotation of L3-level muscle and adipose tissue remains labor-intensive and labeled datasets are scarce.

In parallel with these advances, generic segmentation frameworks such as nnU-Net have demonstrated strong performance in body composition segmentation tasks, including muscle and adipose tissue quantification. nnU-Net automatically configures preprocessing, network architecture, and post-processing for any new dataset without manual intervention, offering a flexible and robust baseline for future method development (77).

#### 5.3.2 Beyond binary segmentation: multi-label parsing

A pivotal advance is the transition from simple muscle-versus-fat segmentation to multi-label fine-grained parsing that simultaneously distinguishes multiple muscle groups and adipose depots. Robert et al. developed such a network. Notably, board-certified radiologists preferred its post-processed segmentations over ground truth annotations in the majority of cases—particularly IMAT, a compartment notoriously difficult to delineate manually (78). This finding suggests that deep learning models may surpass human performance for certain challenging tissue compartments.

#### 5.3.3 Clinical significance of IMAT segmentation

IMAT accumulation within skeletal muscle has emerged as an independent prognostic factor in breast cancer, associated with advanced disease stage and aggressive molecular subtypes. However, manual IMAT segmentation is exceptionally time-consuming and suffers from poor inter-rater reliability, limiting its clinical utility. Paris et al. achieved automated IMAT segmentation within a clinically applicable framework (AutoMATiCA), attaining accuracy approaching human-level performance with millisecond-scale processing speeds (12). By making this tool publicly available, the authors enabled standardized IMAT assessment across research groups—directly addressing a key source of reproducibility concerns.

#### 5.3.4 Open science as a standardization mechanism

A major enabler of methodological standardization has been the open-source release of validated segmentation tools. AutoMATiCA, the multi-label network developed by Robert et al., and the TotalSegmentator-based pipeline developed by Hofmann et al. have all been publicly released with complete code and pre-trained model weights (12, 78, 79). This commitment to open science allows independent research groups to apply identical algorithms to their own datasets, eliminating a key source of between-study variability and facilitating large-scale validation.

In summary, deep learning has transformed CT tissue segmentation from a labor-intensive, operator-dependent manual process into a rapid, reproducible, and increasingly accurate automated workflow. The evolution from fixed thresholds to learned tissue characteristics, from binary segmentation to multi-label parsing, and from closed proprietary tools to open-source frameworks collectively addresses the methodological heterogeneity outlined in Section 2, providing a foundation for standardized, scalable body composition analysis in breast cancer.

### 5.4 Toward standardized outputs: AI-enabled reference systems

Beyond automating existing measurements, AI enables fundamentally new approaches to standardization. Magudia et al. applied a fully automated pipeline to 12,128 outpatients, generating age-, sex-, and race- and ethnicity-specific reference curves for all major body composition parameters (19). This large-scale approach revealed that traditional SMI thresholds introduce significant age- and race-associated bias (P < 0.001), whereas z-scores derived from these reference curves produce consistent sarcopenia prevalence (35%) across all subgroups. The authors further demonstrated that skeletal muscle area z-scores significantly predicted 2-year survival in models that included BMI (P = 0.04), illustrating the clinical value of demographically adjusted metrics.

This paradigm shift—from fixed cutoffs derived from small reference cohorts to dynamic, population-specific reference systems—represents a fundamental advance in sarcopenia diagnosis. Importantly, the rationale for demographically stratified reference curves is not to assert a purely biological basis for racial differences in body composition, but rather to improve diagnostic equity by accounting for the multifactorial determinants—including biological, socioeconomic, and lifestyle factors—that contribute to observed variations across populations. By using z-scores derived from large-scale, demographically diverse reference cohorts, AI-based approaches can mitigate the bias inherent in universal thresholds and reduce the risk of misclassifying sarcopenia in underrepresented groups. AI makes this approach feasible by enabling the large-scale data processing required to establish robust, generalizable reference curves.

### 5.5 Beyond segmentation: multimodal integration and emerging applications

AI’s role extends beyond tissue segmentation. **Table 5** summarizes multimodal approaches that combine body composition parameters with deep learning-extracted radiomic features or clinical data, demonstrating superior predictive performance for metastasis and treatment response.

**Table 5:** Multimodal AI applications in breast cancer body composition research.

Study	Modalities	Prediction target	Performance	Key insight
Qi (2023) (80)	CT body composition + clinical data	Distant metastasis	AUC > 0.96	SHAP analysis identified SMI/T11 cutoff of 21 cm^2^/m^2^; demonstrates data-driven threshold determination
Miao (2022) (81)	T4/T11 CT + deep learning radiomics	Distant metastasis	AUC 0.960	Multimodal fusion of T4 and T11 radiomic features; radiomics complements body composition (Pearson r = 0.115)
Guo (2024) (82)	L3 sarcopenia + MRI radiomics	pCR in TNBC	AUC 0.827	Sarcopenia independently predicts pCR (OR = 0.336); MRI radiomics adds predictive value
Miao (2024) (83)	CT + HOG/LBP features + clinical data	Bone metastasis	AUC 0.922	Multimodal fusion outperforms CT alone (AUC 0.699); subcutaneous fat index is independent predictor
Zhong (2026) (58)	L1 CT body composition + inflammatory/nutritional biomarkers	pCR	AUC 0.888 (internal); 0.831 (test)	SHAP analysis quantifies feature contributions including SMD and VAT density; demonstrates clinical interpretability for individualized decision-making

AUC, area under the curve; CT, computed tomography; HOG, histogram of oriented gradients; LBP, local binary pattern; MRI, magnetic resonance imaging; OR, odds ratio; pCR, pathologic complete response; SHAP, SHapley Additive ExPlanations; SMD, skeletal muscle density; SMI, skeletal muscle index; TNBC, triple-negative breast cancer; VAT, visceral adipose tissue.

Across these studies, several common themes and key differences emerge. SHAP (SHapley Additive ExPlanations) analysis has become the dominant explainability tool, enabling identification of key predictors and data-driven determination of prognostic cutoffs (58, 80). However, the choice of imaging modality and feature extraction strategy varies considerably: some studies focus on single vertebral levels (L3 for sarcopenia, T4/T11 for pectoralis muscle); others employ deep learning radiomics to extract high-dimensional features; and still others adopt a truly multimodal approach combining CT with MRI or integrating imaging data with clinical and laboratory biomarkers (81–83). The predictive targets also differ—ranging from distant metastasis and bone metastasis to pathological complete response—demonstrating the versatility of multimodal AI while highlighting the need for task-specific model design.

Gradient-weighted class activation mapping (Grad-CAM) has identified the erector spinae muscle at T4 and skeletal muscle at T11 as critical regions for metastasis prediction (81, 84). SHAP analysis has enabled data-driven determination of prognostic cutoffs, such as an SMI of 21 cm^2^/m^2^ at T11 for distant metastasis prediction (80). The utility of SHAP extends beyond prognosis to treatment response prediction. Zhong et al. recently demonstrated its application in predicting pCR to neoadjuvant therapy, where SHAP values quantified the contributions of body composition parameters (e.g., SMD, VAT density) alongside inflammatory and nutritional biomarkers, providing individualized decision support (58).

Alternative imaging modalities also benefit from AI integration. Pecchi et al. demonstrated that pectoralis muscle area measured on routine breast MRI correlates strongly with CT-derived body composition parameters and proposed a sarcopenia cutoff of 20.55 cm^2^ with 92% specificity (59). This approach offers a radiation-free alternative for body composition assessment in breast cancer patients undergoing MRI for tumor evaluation.

While these multimodal and explainable AI approaches demonstrate substantial promise, their translation into routine practice faces several challenges—including technical robustness, validation rigor, and regulatory hurdles—which are examined in the following section.

### 5.6 Challenges, limitations, and paths to clinical deployment of AI

The preceding sections have outlined the substantial promise of AI for standardizing CT-based body composition analysis. However, a balanced appraisal requires acknowledgment of the persistent challenges that temper this optimism. Here, we critically examine the technical, validation, regulatory, and integration hurdles that must be overcome before AI-driven body composition profiling can become a routine clinical tool.

#### 5.6.1 Technical robustness and generalizability

The performance metrics reported in the literature—Dice similarity coefficients exceeding 0.95 and sub-second processing times—are typically derived from internal test sets, often using data from a single institution with standardized imaging protocols. The true test of clinical utility lies in generalizability across diverse real-world settings. Domain shift—the degradation of model performance when applied to data from different scanners, reconstruction algorithms, contrast phases, or patient populations—remains a well-documented concern in medical AI (14). For body composition analysis, this is particularly consequential: a model trained on non-contrast, 120 kVp, 5-mm slice CT scans may underperform when applied to contrast-enhanced scans or photon-counting CT systems.

While studies have demonstrated strong correlation between thin-slice and thick-slice automated measurements (49) and linear correction factors for contrast phase have been proposed (48), these adjustments require validation across each new imaging context. Moreover, segmentation failures are non-negligible in challenging cases—anatomical anomalies, severe obesity, motion artifacts, postoperative changes, or metastatic disease distorting normal anatomy—and these are precisely the patients for whom body composition assessment may be most clinically relevant. Reporting only mean Dice scores obscures the distribution of errors; failure cases and their clinical implications must be systematically characterized. Robust evaluation design also requires characterizing model performance under challenging conditions—such as image artifacts and domain shifts—and balancing predictive accuracy with computational efficiency, as demonstrated in lightweight architecture designs for other medical imaging tasks (85).

#### 5.6.2 Annotation standards and model validation

The quality of any AI model is fundamentally bounded by the quality of its training labels. Currently, no universally accepted annotation standard exists for CT-based body composition segmentation. While many studies follow the Alberta Protocol, a systematic review found that 94% of published studies failed to report intravenous contrast use and 63% failed to report slice thickness (3), highlighting the pervasive lack of methodological transparency. Substantial inter-reader variability persists, particularly for challenging compartments such as IMAT, where manual segmentation is notoriously difficult due to complex spatial distribution and subtle attenuation differences (12, 78).

External validation—testing a model on completely independent datasets from different institutions, scanners, and populations—remains the exception rather than the rule (86). Most published AI segmentation tools have been validated internally or on a single external cohort; few have undergone prospective, multi-center validation. Calibration—the alignment between model-predicted probabilities and observed outcomes—is rarely reported, yet it is essential for clinical decision-making, as poorly calibrated models can misguide risk stratification (87, 88).

#### 5.6.3 Interpretability and clinical trust

The transition from technical accuracy to clinical acceptance hinges on interpretability. While Section 3.5 has illustrated how current XAI techniques—such as Grad-CAM for visualizing salient regions and SHAP for quantifying feature contributions—have already been applied to breast cancer body composition research, several critical gaps remain. Visualization-based XAI methods, including saliency maps, feature attribution, and concept-based explanations, have emerged as indispensable tools (89, 90). However, current approaches often lack robustness and reproducibility (90), and the integration of explainability mechanisms into federated and self-supervised learning frameworks remains at an early stage (89).

Beyond the currently applied techniques, a broader set of explainability approaches warrants consideration (91). Uncertainty estimation—quantifying the confidence of each prediction—can alert clinicians to cases where the model is uncertain and manual review is warranted (91, 92). Confidence calibration ensures that predicted probabilities align with true outcome frequencies (92). Counterfactual explanations—answering “what would need to change in the input image for the model to produce a different segmentation or risk prediction?”—offer a powerful mechanism for clinicians to understand model behavior and detect potential failure modes (91, 93). For example, a counterfactual might reveal that a small change in muscle attenuation values would reclassify a patient from sarcopenic to non-sarcopenic, prompting the clinician to question measurement robustness (93).

Ultimately, interpretability is not merely a technical nicety; it is a prerequisite for regulatory approval and clinical adoption. Clinicians must be able to interrogate model outputs, understand failure modes, and exercise appropriate oversight—a paradigm often termed human-AI collaboration rather than AI automation (89). In this paradigm, the model serves as an intelligent assistant that augments, rather than replaces, clinical judgment, with AI outputs providing quantitative data that inform, but do not dictate, clinical decisions.

#### 5.6.4 Regulatory pathways and clinical software certification

For AI-based body composition analysis to be deployed in clinical practice, it must clear regulatory hurdles that vary by jurisdiction. In the United States, the FDA regulates AI/ML-enabled software as medical devices. By 2024, the FDA had authorized 168 ML-enabled Class II devices, of which 94.6% were cleared via the 510(k) pathway and 74.4% were in radiology (94). Recent guidance has enabled the inclusion of Predetermined Change Control Plans (PCCPs), a mechanism that allows sponsors to seek prospective authorization for specified algorithm updates, aligning regulatory review with the rapid cadence of model iteration (94). However, uptake of PCCPs remained limited in 2024 (16.7% of summaries), and only 29.2% of devices reported both sensitivity and specificity (94).

The Good Machine Learning Practice (GMLP) guiding principles, released in 2021 by the FDA, Health Canada, and the UK MHRA, span ten areas including rigorous software engineering, representative datasets, human-AI team performance, and post-deployment monitoring (94). In Europe, the EU AI Act classifies diagnostic support tools as high-risk AI systems, requiring conformity assessment and CE marking. These regulatory frameworks demand rigorous clinical validation, ongoing performance monitoring, and post-market surveillance—requirements that few research-grade AI tools have met. The path from a published algorithm to a certified clinical product is long and costly, involving not only technical refinement but also prospective clinical trials, quality management systems, and continuous model maintenance to guard against performance drift over time.

Encouragingly, some body composition analysis tools have already entered the regulatory pathway. DAFS (Data Analysis Facilitation Suite) received FDA 510(k) clearance in March 2026, indicating that AI-based CT body composition analysis is transitioning from research to regulated clinical products (95). Similarly, MuscleView—an AI tool for automated segmentation of musculoskeletal structures from MRI—received 510(k) clearance in October 2024, demonstrating the regulatory feasibility of AI-based muscle quantification (96).

#### 5.6.5 Integration into clinical workflows

Even a perfectly performing AI algorithm has no clinical value if it cannot be seamlessly integrated into existing infrastructure. From a technical perspective, frameworks such as MONAI Deploy support the integration of AI pipelines into existing imaging networks by providing standardized interfaces for DICOM input and output, allowing AI models to operate within routine radiology workflows without requiring custom infrastructure (97). However, the broader operational requirements for successful clinical deployment—including PACS/RIS integration, structured reporting, quality control and human oversight, and prospective multi-center validation.

**Figure 3:**
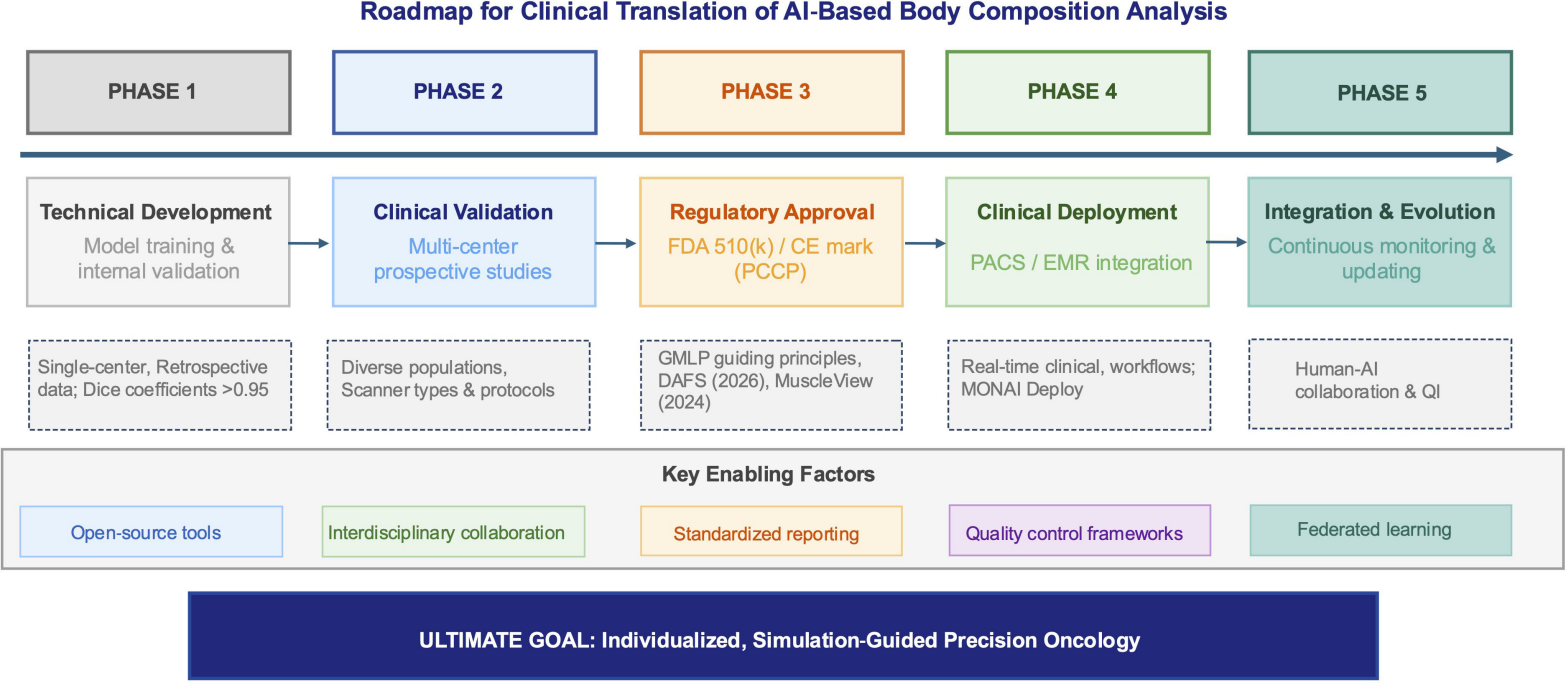
Roadmap for clinical translation of AI-based body composition analysis. The pathway comprises five sequential phases toward integrating AI-driven body composition profiling into routine oncology practice. Phase 1: technical development, including model training and internal validation on retrospective datasets, where current platforms have achieved Dice coefficients exceeding 0.95. Phase 2: prospective multi-center clinical validation across diverse populations, scanner types, and imaging protocols. Phase 3: regulatory approval, including FDA 510(k) or CE marking, with consideration of Predetermined Change Control Plans (PCCPs) and Good Machine Learning Practice (GMLP) guidelines; DAFS (2026) and Muscle View (2024) are recent examples of regulatory clearance. Phase 4: clinical deployment, integrating AI pipelines into PACS and electronic medical records (e.g., via MONAI Deploy). Phase 5: continuous performance monitoring, model updating, and human-AI collaboration with quality improvement mechanisms. Key enabling factors include open-source tools, interdisciplinary collaboration, standardized reporting, quality control frameworks, and federated learning. The ultimate goal is individualized, simulation-guided precision oncology. PACS, picture archiving and communication system; EMR, electronic medical record; PCCP, Predetermined Change Control Plan; GMLP, Good Machine Learning Practice.

In summary, AI offers a powerful pathway toward standardization of CT-based body composition analysis, but its clinical translation is contingent on addressing substantial challenges in technical robustness, validation rigor, interpretability, regulatory certification, and workflow integration. Recognizing these limitations is not to diminish AI’s promise but to chart a realistic course toward its responsible and effective deployment. **Figure 3** presents a roadmap for clinical translation, outlining five sequential phases from technical development to routine clinical deployment and continuous model evolution.

## 6 Discussion

CT-derived body composition parameters are robust prognostic and predictive biomarkers in breast cancer, yet their clinical translation has been hindered by substantial methodological heterogeneity. The fragmentation of diagnostic thresholds—particularly for sarcopenia, myosteatosis, and visceral obesity—prevents reliable application of research findings to individual patients and impedes meaningful cross-study comparisons.

Artificial intelligence, particularly deep learning, offers a promising solution. By enabling fully automated, standardized analysis across the entire workflow—from slice localization and tissue segmentation to reference curve generation and multimodal risk prediction—AI addresses each source of variability identified in this review. Critically, AI does not merely automate existing imperfect methods; it enables fundamentally new approaches to standardization, such as population-specific reference curves and data-driven threshold determination.

### 6.1 Limitations

This narrative review has several limitations. First, as a non-systematic review, it may be subject to selection bias, and no quantitative synthesis was performed. Second, the rapid pace of AI evolution means that some emerging architectures may not have been fully captured. Third, although we focused on breast cancer, the generalizability of our proposed standardization framework to other cancer types requires further validation. Despite these limitations, the methodological heterogeneity framework we describe remains broadly applicable.

### 6.2 Future directions and clinical implementation

Several priorities emerge from this review. Prospective multicenter studies are needed to validate AI-based body composition analysis across diverse populations, scanner types, and clinical protocols. Consensus guidelines should be developed for standardized thresholds, with particular attention to breast cancer-specific and population-specific cutoffs. Regulatory pathways for AI-assisted diagnostic software should be pursued to enable clinical deployment.

### 6.3 Bridging the implementation gap: from algorithm to clinical workflow

Technical validation alone does not ensure clinical adoption. Several operational requirements must be addressed before AI-driven body composition profiling can be embedded into routine practice. The challenges of translating algorithm development into clinical workflow integration—including real-time constraints, computational demands, and user interface design—have been well documented in medical imaging (98).

First, PACS/RIS integration is a prerequisite for seamless deployment. A PACS-integrated AI workflow has been shown to achieve high technical success rates with near-real-time processing, enabling opportunistic screening without disrupting existing workflows (99, 100). Frameworks such as MONAI Deploy further support this integration by providing standardized interfaces for DICOM input and output (97).

Second, structured reporting using DICOM Structured Reporting (SR) standards allows AI-generated metrics—including muscle area, fat areas, and attenuation—to be embedded directly into radiology reports and made accessible to clinicians via electronic medical records (101).

Third, quality control and human oversight remain essential. AI segmentation can fail in cases of anatomical anomalies, severe obesity, or postoperative changes. A human-in-the-loop mechanism—allowing radiologists to accept, reject, or request correction of AI outputs—is necessary to ensure patient safety and maintain clinical trust (101). These governance questions have no universally accepted answers and must be addressed institution by institution.

Fourth, prospective, multicenter validation in real-world clinical settings is critical to demonstrate generalizability (102). These operational considerations, though less frequently highlighted in technical literature, are as important as algorithmic performance for successful clinical translation.

### 6.4 Multimodal integration and digital twins

Multimodal integration extends beyond CT-only analysis. By combining imaging data with clinical variables, multimodal deep learning models can enhance predictive performance. Alipour et al. developed XComposition, which estimates CT-based body composition metrics from chest radiographs and four clinical variables in a cohort of 1,118 patients (Pearson r = 0.85 for subcutaneous fat, 0.76 for visceral fat). The multimodal late-fusion strategy significantly outperformed unimodal models, demonstrating that combined modalities capture complementary information (103). This approach suggests that widely available imaging modalities could enable large-scale opportunistic screening in populations such as early-stage breast cancer patients who undergo chest imaging only.

This opportunistic screening approach addresses the immediate challenge of data accessibility. Looking beyond, digital twins—dynamic, patient-specific models that simulate disease trajectories and treatment responses—represent a more transformative paradigm (104). Wu et al. demonstrated their feasibility in triple-negative breast cancer, constructing MRI-based digital twins for 105 patients receiving neoadjuvant chemotherapy. The framework accurately predicted pathological complete response (AUC 0.82) and identified patient-specific regimens that could improve response rates by over 20% (104). While this proof-of-concept study focused on tumor response rather than body composition, it illustrates the broader potential: integrating serial CT-derived muscle and fat metrics with tumor genomics could enable prospective simulation of how host composition changes influence treatment tolerance and efficacy.

Looking beyond quantitative analysis, the integration of multimodal large language models (MLLMs) into radiology workflows is an emerging frontier. MLLMs have shown promise in tasks such as structured report generation, visual question answering, and interactive decision support by combining imaging data with clinical text and electronic health records (105, 106). While their direct application to CT body composition analysis remains largely unexplored, these models could eventually facilitate the integration of quantitative body composition metrics into narrative clinical reporting and multidisciplinary decision-making.

Finally, interdisciplinary collaboration among radiologists, oncologists, nutritionists, and data scientists is essential to translate technical advances into improved patient outcomes. Structured body composition reports should be integrated into multidisciplinary tumor boards to guide chemotherapy dosing, surgical risk stratification, and nutritional interventions.

AI provides a foundation for standardized, scalable, and clinically actionable body composition profiling, although substantial challenges in validation and deployment remain. The transition from fixed, population-derived thresholds to dynamic, individualized reference systems exemplifies how AI can fundamentally transform biomarker development. As the field matures, integrating automated body composition analysis into routine clinical workflows promises to advance precision oncology for breast cancer patients worldwide.
